# Enantiomeric Tartaric Acid Production Using *cis*-Epoxysuccinate Hydrolase: History and Perspectives

**DOI:** 10.3390/molecules24050903

**Published:** 2019-03-05

**Authors:** Jinsong Xuan, Yingang Feng

**Affiliations:** 1 Department of Biological Science and Engineering, School of Chemical and Biological Engineering, University of Science and Technology Beijing, 30 Xueyuan Road, Beijing 100083, China; 2 Shandong Provincial Key Laboratory of Synthetic Biology and CAS Key Laboratory of Biofuels, Qingdao Institute of Bioenergy and Bioprocess Technology, Chinese Academy of Sciences, Songling Road 189, Qingdao, Shandong 266101, China

**Keywords:** *cis*-epoxysuccinate hydrolase, tartaric acid, enantioselectivity, stereoselectivity, regioselectivity, epoxide hydrolase, immobilization, whole cell catalyst, enzyme stability, biocatalyst

## Abstract

Tartaric acid is an important chiral chemical building block with broad industrial and scientific applications. The enantioselective synthesis of l(+)- and d(−)-tartaric acids has been successfully achieved using bacteria presenting *cis*-epoxysuccinate hydrolase (CESH) activity, while the catalytic mechanisms of CESHs were not elucidated clearly until very recently. As biocatalysts, CESHs are unique epoxide hydrolases because their substrate is a small, mirror-symmetric, highly hydrophilic molecule, and their products show very high enantiomeric purity with nearly 100% enantiomeric excess. In this paper, we review over forty years of the history, process and mechanism studies of CESHs as well as our perspective on the future research and applications of CESH in enantiomeric tartaric acid production.

## 1. Introduction

Tartaric acid (TA) is a well-known organic acid that naturally occurs in many kinds of fruit, most notably in grapes. The chemical chirality of TA was first discovered by Jean-Baptiste Biot in 1832 [1]. The naturally-occurring form of the acid is l(+)-TA, while d(−)-TA rarely exists in natural sources [2,3]. l(+)-TA is widely used in the food, wine, pharmaceutical, chemical, and polyester industries. d(−)-TA is also important in pharmaceutical manufacturing [4,5,6]. Both are well-known chiral chemical building blocks with broad industrial and scientific applications [7,8]. In enantioselective chemical synthesis, TA serves not only as a resolving agent or chiral auxiliary in the synthesis of bioactive molecules, but also a source of new asymmetric organocatalysts [7,8,9]. Traditionally, l(+)-TA is obtained as a solid by-product during wine fermentation, and this kind of production method is strongly influenced by the growth of grapes and the climatic conditions. Chemical synthesis of l(+)-TA with maleic acid is also possible but this gives a much less soluble racemic product (DL-form) which is not suitable for inclusion in foods because d(−)-TA in the product is considered to be harmful to human health. Commercial application of the chemical method is limited by both the product form and the high production cost [10]. Currently, microbial methods are considered to be much simpler and more economical for the production of l(+)-TA and d(−)-TA.

Epoxide hydrolases (EHs, EC 3.3.2.3) are biocatalysts that are ubiquitous in Nature. They can hydrolyze racemic epoxides to their corresponding optically active epoxides and pure vicinal diols, which are versatile intermediates for chiral pharmaceutical synthesis. In general, this enzymatic process occurs under mild conditions without the need for any cofactors, prosthetic groups, or metal ions [11]. The high transformation rate and enantioselectivity of epoxide hydrolases have gained them increasing attention in recent years, and they have found more and more applications in the organic chemical industry [11]. Epoxide hydrolases are found in a variety of sources, such as plants, insects, mammals, and microbes [12,13,14]. Mammalian epoxide hydrolases have been the subject of many studies because of their key role in xenobiotic detoxification in the liver, but their use as biocatalysts has been hindered by their limited availability [15]. Lately, bacterial epoxide hydrolases have been increasingly recognized as highly versatile biocatalysts owing to their abundance, high efficiency, and environmental friendliness [16].

*cis*-Epoxysuccinic acid hydrolases (CESHs) are epoxide hydrolase members that catalyze the asymmetric hydrolysis of *cis*-epoxysuccinate (CES) to form an enantiomeric tartrate [17,18,19]. Bacteria presenting CESH activity were discovered in the 1970s, and the synthesis of l(+)-TA was the first application of an epoxide hydrolase [20]. Since then, a large number of bacteria with CESH activity have been discovered and successfully applied for industrial TA production. The sequences and mechanism of CESHs have been partly elucidated since 2000, when it was revealed that CESH[L] and CESH[D], which produce l(+)-TA and d(−)-TA, respectively, are completely different proteins in terms of both sequence and structure. Therefore, CESHs are interesting EHs not only for TA production, but also for enantiomer biosynthesis in general. In this review, we summarize the body of literature on CESHs including both process optimization for industrial application and mechanism studies to understand how their regio- and stereoselectivity makes them efficient biocatalysts. We also provide our perspective on the use of CESHs in future research and applications.

## 2. The History of CESH Studies

Several patents involving bacteria with CESH activity were filed by Japanese companies in the 1970s [21,22,23,24,25,26]. However, these bacteria and their associated CEHSs did not receive much attention in the following ten years. In fact, unlike mammalian EHs, which have been subject to extensive enzymatic and biochemical study, microbial EHs were not well studied until the 1990s. Several studies reported in the 1990s showed that bacterial CESHs are promising biocatalysts for industrial synthesis, and EHs were proposed as new tools for the synthesis of fine organic chemicals [16,20]. In the 1990s, several groups from China, Belgium, Japan, and Slovakia continued to report on new bacteria containing CESH activity, and some process optimization methods, such as immobilization, were performed for industrial application [3,10,27,28,29,30,31]. The microbial production of L(+)-tartaric acid was successfully commercialized in the late 1990s [32]. Subsequently, new bacterial species were isolated for TA production in the 21st century [18,33,34,35,36,37,38,39], which demonstrates that CESHs exist broadly in bacteria. 

In 2000, the first CESH encoding gene was reported from *Alcaligenes* sp. MCI3611 for the production of d(−)-TA [40]. Genes from several other CESHs, including CESH[D] from *Bordetella* sp. BK-52 and CESH[L]s from *Rhodococcus opacus*, *Nocardia tartaricans*, and *Klebsiella* sp. BK-58, have also been sequenced and cloned [17,19,41,42,43]. The genes and the derived amino acid sequences of CESHs provided the basis for later studies of the recombinant expression, structure, and mechanisms as well as the protein engineering of CESHs. Analyses of the amino acid sequences of CESHs indicated that CESH[D]s and CESH[L]s are completely different proteins [19]. Subsequent structural analysis and mechanism studies revealed that they have different structures and catalysis mechanisms [41,43,44,45]. In a recent study [46], we determined a high-resolution structure and elucidated detailed catalytic mechanisms for CESH[D], but these characteristics have not yet been reported for CESH[L].

With the knowledge of CESH sequences, structures, and mechanisms, scientists began to conduct extensive protein engineering research to improve enzyme stability and activity. As a result, many mutants with good properties have been obtained for potential industrial use [42,47,48]. However, these studies were accomplished without knowledge of the high-resolution structures of CESHs; therefore, there is potential to further improve the use of CESHs as biocatalysts in industrial applications.

## 3. Bacteria that Produce CESHs

CESHs catalyze the enzymatic hydrolysis of *cis*-epoxysuccinate to form l(+)-TA or d(−)-TA with high product enantioselectivity. TA products obtained by the hydrolysis of CES using purified CESHs generally have enantiomeric excess (EE) values of near 100% [19,36,38,39,44,45]. Therefore, bacteria that produce CESH[D] or CESH[L] have been separately reported. More than twenty species have been isolated by researchers and are distributed among more than ten genera (Table 1). Isolated species with CESH[L] activity include both Gram-positive and Gram-negative bacteria, while to date, all species with CESH[D] activity are Gram-negative. Only the genus *Pseudomonas* has both type of species. 

More than ten species with CESH[L] activity have been reported since the 1970s. Miura et al. discovered that microorganisms of the genus *Nocardia* can produce CESH[L]; specifically *Nocardia tartaricans nov. sp.* was identified as a preferred natural species [24]. Kamatani et al. also isolated microorganisms capable of hydrolyzing *cis*-epoxysuccinate to l(+)-TA belonging to the genera *Pseudomonas*, *Agrobacterium*, and *Rhizobium* [22]. Two strains of *Rhizopus validum* and *Corynebacterium* JZ-1 that were discovered in soil can produce l(+)-TA, and the latter has a molar conversion rate of *cis*-epoxysuccinate as high as 96% [27,28]. Screening of 65 *Nocardia* strains identified *Nocardia* sp. SW 13-57 as a high-yield strain with the ability to produce CESH[L]. Its molar conversion rate is over 90% and the CESH[L] formation is effectively induced by sodium *cis*-epoxysuccinate during fermentation [29]. In addition, *Rhodococcus ruber* M1 isolated from soil was the first strain in *Rhodococcus* reported to produce CESH[L] [34]. Further, a strain of *Klebsiella* sp. BK-58 can produce a novel CESH[L] with good thermal and pH stability, which is a promising biocatalyst for the industrial production of l(+)-TA [38].

Ten species in four genera have been reported to have CESH CESH[D] activity. Sato et al. first isolated four novel species of *Achromobacter* and two novel species of *Alcaligenes* with CESH[D] activity [21]. A strain belonging to the genus *Pseudomonas* and the microbial cells of *Alcaligenes* sp. MCI3611 also has the capability to hydrolyze *cis*-epoxysuccinate to d(−)-TA [31,33]. The DNA fragment encoding the enzyme to produce d(−)-TA was successfully obtained from the chromosomal DNA library of *Alcaligenes* sp. MCI3611 [40]. Two strains from the genus *Bordetella* (*Bordetella* sp. strain 1-3 and *Bordetella* sp. BK-52), isolated from vegetable fields in Hangzhou, were able to transform *cis*-epoxysuccinate to d(−)-TA [35,36]. Unlike traditional *Bordetella* species that are exclusively associated with humans and warm-blooded animals, both of these strains are from the natural environment. Unlike previously reported CESH[D] producing bacteria, *Bordetella* sp. strain 1-3 has also been reported to have the ability to degrade d(−)-TA as its carbon source, so some measures should be adopted to stop this degradation process to accumulate d(−)-TA. Furthermore, the molecular weight of CESH[D] from *Bordetella* sp. strain 1-3 is the same as the beta subunit of the previously reported CESH[D] from *Alcaligenes* sp. The eight amino acid sequence of the N-terminal region of CESH[D] from *Bordetella* sp. strain 1-3 has also been shown to have the same sequence as the beta subunit from *Alcaligenes* sp. [19].

The gene sequences of several CESH-producing bacteria have been reported, including CESH[L] genes from *Rhodococcus opacus*, *Nocardia tartaricans* CAS-52, and *Klebsiella* sp. BK-58, and CESH[D] genes from *Alcaligenes sp.* MCI3611 and *Bordetella* sp. BK-52 [17,19,37,40,43]. Some of these genes have been successfully expressed in *Escherichia coli* [17,19,37,43]. The amino acid sequences of CESH[L] from *Rhodococcus opacus* and *Nocardia tartaricans* CAS-52 are identical, but they only share 36% sequence identity with the CESH[L] from *Klebsiella* sp. BK-58. The two CESH[D]s from *Alcaligenes* sp. MCI3611 and *Bordetella* sp. BK-52 have identical amino acid sequences. The amino acid sequences of CESHs from most bacteria in Table 1 are still unknown.

## 4. Stability of CESHs

Although CESHs have excellent product enantioselectivity and high activity, pure CESHs are unstable and heat-sensitive and are thus unsuitable for industrial applications [3,17,49]. A continuous bioconversion study using *Rhodococcus rhodochrous* showed that the effect of the large increase in stability at a lower temperature was much more important than the decrease in activity [3]. To improve the stability of CESHs at the optimal pH and temperature, whole-cell immobilization was adopted for the industrial bioconversion process. Carriers including gelatin beads, pectate gel beads, and κ-carrageenan were screened, and the process of immobilization was optimized for different species [30,49,50,51,52,53,54]. Whole-cell immobilization was shown to not only increase the stability of the biocatalysts, but also improve the activity and conversion ratio.

CESH[L] and CESH[D] show different stabilities. CESH[D] from *Bordetella* sp. BK-52 has high stability and activity in a broad range of temperatures (37–45 °C) and pH values (4.6–9.0) with optimal conditions being 40 °C and pH 6.5 [19]. A comparison study indicated that CESH[D] has greater thermal and pH stability than CESH[L] [41]. However, the recently discovered novel CESH[L]s from *Klebsiella* sp. and *Labrys* sp. BK-8 have good thermal and pH stability. The former is stable up to 50 °C and at pH 5 to 11, while the latter is stable over a broad range of temperatures and pH values with the greatest activity occurring at 50 °C and 8.5 [38,39]. Therefore, they could be used as alternative biocatalysts for the production of l(+)-TA.

In addition, the stability and activity of CESHs can also be improved by protein engineering methods such as fusing a binding module to CESH or changing the protein primary structure. For example, the wild-type CESH[L] gene from *Rhodococcus opacus* has been fused with a carbohydrate binding module (CBM30), and the resulting fusion enzyme (CBM30-CESH) exhibited improved temperature and pH adaptability than free native CESH[L] [55]. The CESH[L] mutant 5X-1 from *Rhodococcus opacus* was successfully constructed by combining directed evolution with various semi-rational redesign methods. The optimal reaction temperature using mutant 5X-1 occurred at 55 °C, which is much higher than the optimal temperature at 35 °C using the wild-type enzyme. The pH range for the effective working of mutant 5X-1 extended from 8.0–9.0 to 5.0–10.0 [47]. Random mutation by error-prone PCR and high throughput screening revealed that single point mutations on the Phe10 residue of CESH[L] from *Klebsiella* sp. BK-58 resulted in different levels of enzyme thermostability and catalytic activity. The mutant F10Q had 230% higher activity but lower stability than the wild-type enzyme [48].

## 5. Process Optimization for TA Production Using CESHs

Since the discovery of CESH activity in the 1970s, CESH-producing bacteria and CESHs have been utilized to produce TA with high enantiopurity. The cell lysate or crude enzyme solution is not suitable for TA production because the cellular protease degrades CESH rapidly. Therefore, significant effort has been made to optimize TA production using CESHs or CESH-producing bacteria. TA production was established in the 1970s including the surfactant addition and recovery of TA from the media [23,25]. Subsequent studies have found that these enzymes have low stability [3], so studies on process optimization have mainly focused on the methods of immobilization and recombination. 

Bacterial cells can be immobilized by different carriers and show different levels of efficiency and stability. Different microorganisms have different optimal immobilization methods, for example, the best cell immobilization carriers for *Nocardia tartaricans*, *Corynebacterium*, and *Rhizobium* are gelatin, κ-carrageenan, and sodium alginate, respectively [30,52,56]. The immobilization of *Labrys* and recombinant *E. coli* cells with carrageenan is also an excellent process for TA production with high efficiency and stability [39,54]. Aside from the carriers, the subsequent processes of immobilization also have important effects on both the activity and stability. Rosenberg et al. found that although κ-carrageenan is an excellent carrier for the immobilization of *Nocardia tartaricans* cells, the use of cross-linked calcium pectate gel (CPG) is advantageous for the preparation of spherical particles with high activity and stability [50,51]. Sodium alginate–cellulose sulfate-poly(methylene-*co*-guanidine) (SA-CS/PMCG) capsules have been shown immobilize *Nocardia tartaricans* with a better performance than CPG [49]. Additionally, various surfactants can greatly enhance the activity of the immobilized cells, mainly through a change in the permeability of the cell membrane [50,52,57]. Dong et al. reported that ultrasound treatment could be used to change the cell permeability and improve the bioconversion efficiency of immobilized *E. coli* cells containing expressed recombinant CESH[D] [58].

The genes of CESH[L] and CESH[D] have been cloned and expressed in *E. coli* successfully [17,19,40,41,43]. Therefore, recombinant CESHs also have good potential to be used in industrial TA production. Some studies have reported that the stability of enzymes can be improved by immobilization. For example, we significantly improved the stability of CESH[L] by fusing it with CBM [55], and then purified and immobilized it on cellulose in one step. Wang et al. immobilized CESH[L] on agarose Ni-IDA to enhance its stability [59]. The preparation of recombinant CESH[L] was also improved by the utilization of the heat-induced promoter to avoid the chemical induction of protein expression [60]. Still, more effort is needed to optimize the stability of recombinant CESH and the process of TA production using the recombinant enzyme. 

## 6. Structure and Catalytic Mechanism of CESH[L]

Until now, there have been no reports on the structure of CESH[L]. Only two CESH[L] sequences have been reported and they share a 36% sequence identity [17,37,43]. Both have about a 30% sequence identity with l-2-haloacid dehalogenase, of which the structure is known. Therefore, homology modeling has been performed to elucidate the catalytic mechanism of CESH[L] [41,42,44]. L-2-haloacid dehalogenase has an α/β hydrolase fold that is adopted by most EHs [61]. Therefore, it is likely that CESH[L] also adopts the α/β hydrolase fold, and the catalytic mechanism of CESH[L] has been proposed to be similar to most EHs, i.e., a two-step mechanism including an ester intermediate [61]. The two-step mechanism was confirmed by ^18^O experiments for both CESH[L]s [43,44]. 

As CESH[L] and L-2-haloacid dehalogenase only have about a 30% sequence identity and different substrates, the catalytic residues cannot be deduced from homology modeling. Two mutagenesis analyses revealed that D18, H190, and D193 are essential for the activity; therefore, they were proposed to be a catalytic triad, with D18 activating the attacking water molecule [42,44]. However, as the CES substrate is a polar hydrophilic molecule, the active site of CESH[L] contains many charged and hydrophilic residues, making it difficult to elucidate the role of each residue without a high-resolution crystal structure of the CESH[L]-substrate complex. The proton donor that may facilitate the ring opening is still not clear, and how the CES is fixed in the active site to ensure the stereoselectivity also remains unknown.

## 7. Structure and Catalytic Mechanism of CESH[D]

Although CESH[D] and its gene sequence were reported earlier than CESH[L], knowledge of CESH[D] catalysis was not obtained until very recently. The protein sequences of CESH[D]s from *Bordetella* sp. BK-52 and *Alcaligenes* sp. MCI3611 have about 30% sequence identity to the Kce enzyme, whose function is totally different [19,40,41]. Homology modeling using Kce as a template indicated that CESH[D] has a TIM barrel fold with a metal ion which is crucial for its activity [41,45]. The metal ion was identified to be a divalent ion, either zinc, calcium, or magnesium [41,45], which is coordinated by three residues in Kce; however, only two of these are conserved in CESH[D] [41,45]. The third coordinative residue could not be identified before the CESH[D] structure was determined. An ^18^O labeling experiment indicated that CESH[D] hydrolyzes CES through a one-step mechanism [45] instead of the two-step mechanism of CESH[L]. As CESH[D] and Kce have different substrates, their active sites are very different and their catalytic residues cannot be deduced by homology modeling. Mutagenesis studies have identified a large number of essential residues, so it is difficult to identify the key catalytic residue and stereoselective catalytic mechanism from these studies without a high-resolution CESH[D] structure. 

The catalytic mechanism of CESH[D] was not elucidated in detail until our recent report on high-resolution CESH[D] structures [46]. The structure of substrate-free CESH[D] revealed not only the third metal coordinative residue (Glu14), but also three coordinative water molecules that formed an equilateral triangle. Trials using an inactive mutant and CES co-crystallization obtained an unexpected CESH[D] structure in complex with its reaction product, d(−)-TA. In the complex structure, three oxygen atoms of TA occupy the positions of the three coordinative water molecules in the substrate-free CESH[D] structure. The identification of the structure of the product–enzyme complex provided the details of substrate binding and positioning, from which the key catalytic residues and substrate recognition residues were elucidated. Instead of the previously proposed catalytic residue D251 [45], the catalytic residues were identified as D115 and E190, while R11 provided the proton and facilitated the ring opening. D251 played a crucial role in fixing the position of R11, which supports the importance of this residue, as identified in the previous mutagenesis analysis. This structure and catalytic mechanism explained the stereoselectivity, regioselectivity, and substrate selectivity of CESH[D] [46]. 

**Figure 1:**
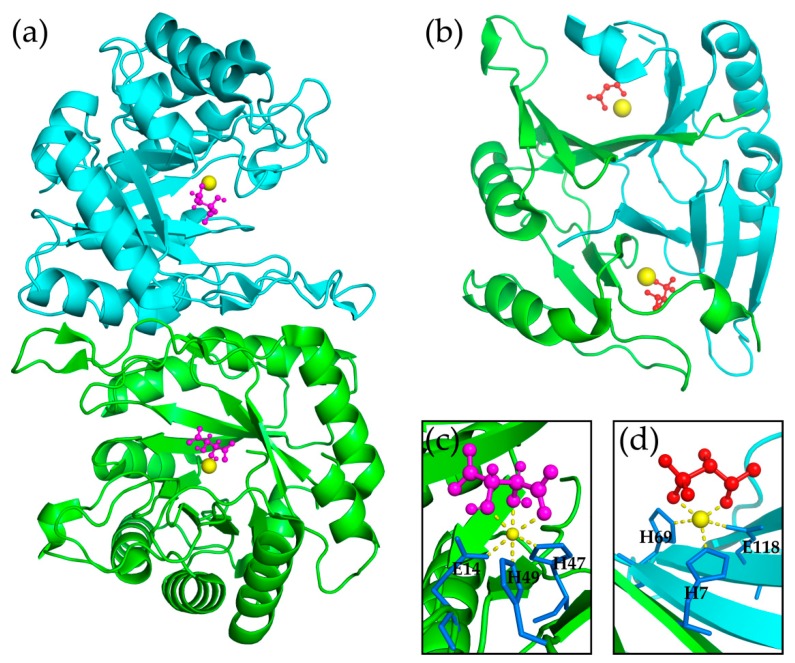
Comparison of CESH[D] and FosX: (**a**) structure of CESH[D]; (**b**) structure of FosX; (**c**) the active site of CESH[D]; (**d**) the active site of FosX. Both proteins are dimeric and colored in green and cyan for each monomer. The metal ions are shown as yellow balls. The products are shown as magenta and red ball and sticks for CESH[D] and FosX, respectively. In (**c**,**d**), the three coordinative residues of the metal ion are shown as blue sticks.

The structure of CESH[D] has some distinct features in comparison with known EHs. In contrast with the α/β hydrolase fold or LEH fold adopted by most EHs, except for LA4H, ChEH, and FosX, CESH[D] adopts a TIM-barrel fold [61]. Also, CESH[D] has a one-step mechanism, while α/β hydrolase fold EHs have a two-step mechanism. LEH adopts a one-step mechanism, but the substrate specificity is determined by the hydrophobic interaction (molecular shapes), and no metal ion is needed for LEH [62]. The EH with the most similar mechanism is FosX, which also contains a metal ion for substrate binding and adopts a one-step mechanism (Figure 1) [63]. However, FosX has a dimeric VOC family fold with paired βαβββ where the active sites are located at the dimer interface, and the substrate of FosX only occupies two coordination sites of the metal ion with square pyramidal coordination geometry [63]. Although CESH[D] is also a dimeric protein, its active sites are located at the center of each dimer’s subunit, and its metal elements have octahedral coordination geometry where three of the coordination sites are occupied by the substrates [46]. Therefore, CESH[D] is a unique EH in terms of both its protein fold and catalytic mechanism. 

## 8. Perspective for CESH Research and Application

CESHs are unique among the known EHs because the CES substrate is highly hydrophilic and mirror-symmetric. The structural features of CES suggest that CESHs have specific substrates; in other words, the CES molecule is fixed in CESH with an exact position, which leads to the high stereoselectivity and regioselectivity of CESHs. This feature is of great interest for enantioselective synthesis. Although CESHs and their host bacteria have been successfully applied in industry for TA production, there are still many questions to be addressed, and there is a lot of room for growth in the production of TA using CESHs. 

Currently, the structure and the catalytic mechanism of CESH[L] are still not fully understood. Determination of the high-resolution structure of CESH[L], particularly of complexes with a substrate or product, is necessary to elucidate its catalytic mechanism. With this structure, rational engineering to enhance the stability will be possible. Furthermore, CESHs could also potentially be engineered as biocatalysts to catalyze different substrates, but this potential has not been explored in past studies. 

Although many microorganisms have been reported to have CESH activity, only very few of them have been sequenced. Therefore, much work is still needed to isolate the CESHs and analyze their protein/gene sequences. The new sequences of CESHs may provide other new features that will help us to understand these enzymes and promote their applications.

Currently, the production of TA using whole cell catalysts is performed by wild strains. No metabolic engineering of these microorganisms has previously been reported. Therefore, understanding the features of these microorganisms and the development of genetic engineering are important topics for future studies. CESHs are intracellular enzymes that are present in certain microorganisms, which cause the activity of whole cell catalysts to depend on the cell permeability. If the CESHs could be engineered as a secretive protein, or immobilized on the cell surface, their activity would be greatly enhanced. This engineering could be done in either the original species or the recombinant *E. coli* cells, and it will improve the production of enantiomerically pure TA using CESHs. 

## Figures and Tables

**Table 1:** Strains producing *cis*-epoxysuccinic acid hydrolases (CESHs).

CESH	Genus	Species	Gene	Reference
CESH[L]	*Acetobacter*	*Acetobacter curtus*		[26]
	*Acinetobacter*	*Acinetobacter tartarogenes*		[23]
	*Agrobacterium*	*Agrobacterium aureum*		[22]
		*Agrobacterium viscosum*		[22]
	*Corynebacterium*	*Corynebacterium* S-13		[26]
		*Corynebacterium* sp. JZ-1		[28]
	*Klebsiella*	*Klebsiella* sp. BK-58	KF977193	[38,43]
	*Labrys*	*Labrys sp.* BK-8		[39]
	*Nocardia*	*Nocardia tartaricans*		[24]
		*Nocardia tartaricans* SW13-57		[29]
		*Nocardia tartaricans* CAS-52	JQ267565	[37]
	*Pseudomonas*	*Pseudomonas* sp. KB-86		[22]
	*Rhizobium*	*Rhizobium validum*		[22,27]
	*Rhodococcus*	*Rhodococcus opacus*	DQ471957	[17]
		*Rhodococcus ruber* M1		[34]
		*Rhodococcus rhodochrous*		[3]
CESH[D]	*Achromobacter*	*Achromobacter tartarogenes*		[21]
		*Achromobacter epoxylyticus*		[21]
		*Achromobacter acinus*		[21]
		*Achromobacter sericatus*		[21]
	*Alcaligenes*	*Alcaligenes epoxylyticus*		[21]
		*Alcaligenes margaritae*		[21]
		*Alcaligenes* sp. MCI3611	^1^	[33,40]
	*Bordetella*	*Bordetella* sp. strain 1–3		[35]
		*Bordetella* sp. BK-52	EU053208	[19,36]
	*Pseudomonas*	*Pseudomonas putida*		[31]

^1^ The gene and protein sequence were reported in a patent [40].
